# $L_h^2$-Functions in Unbounded Balanced Domains

**DOI:** 10.1007/s12220-016-9754-3

**Published:** 2017-01-02

**Authors:** Peter Pflug, Włodzimierz Zwonek

**Affiliations:** 1 Institut für Mathematik, Carl von Ossietzky Universität Oldenburg, Postfach 2503, 26111 Oldenburg, Germany; 2 Faculty of Mathematics and Computer Science, Institute of Mathematics, Jagiellonian University, Łojasiewicza 6, 30-348 Kraków, Poland

**Keywords:** Balanced domain, Lelong number, Bergman space, $L_h^2$-functions, $L_h^2$-domain of holomorphy, 32A07, 32A36, 32D05, 32U25

## Abstract

We investigate problems related with the existence of square integrable holomorphic functions on (unbounded) balanced domains. In particular, we solve the problem of Wiegerinck for balanced domains in dimension two. We also give a description of $L_h^2$-domains of holomorphy in the class of balanced domains and present a purely algebraic criterion for homogeneous polynomials to be square integrable in a pseudoconvex balanced domain in $\mathbb {C}^2$. This allows easily to decide which pseudoconvex balanced domain in $\mathbb {C}^2$ has a positive Bergman kernel and which admits the Bergman metric.

## Introduction

As a starting point for the considerations presented in the paper, the following problem of Wiegerinck from 1984 (see [24]) may serve. Does the Bergman space $L_h^2(D):=L^2(D)\cap \mathcal {O}(D)$ allow its dimension to be only either 0 or $\infty$ for an arbitrary pseudoconvex domain $D\subset \mathbb {C}^n$? Wiegerinck confirmed the dichotomy in dimension one (see also [23]) and gave an example of an unbounded non-pseudoconvex domain $D\subset \mathbb {C}^2$ with non-trivial $L_h^2$-functions but having its space $L_h^2(D)$ finitely dimensional. One of the main results of the present paper is Theorem 7 in which the conjecture is confirmed for the class of balanced domains in dimension two. Moreover, in the same class of domains, we present a complete description of domains admitting no non-trivial $L_h^2$-functions answering partially a question posed in [8] and [22] about the characterization of such domains in arbitrary dimension. Let us also mention that a recent result on the infinite dimensionality of $L_h^2(D)$ (see [7]) in a wide class of domains cannot be applied in the general case in our situation because the so-called core of a domain may be equal to the whole domain (belonging to the Siciak class of domains—definition below, see also the discussion in Sect. 3.1).

Basic objects of study in several complex variables are domains of holomorphy defined as follows: For the family $\mathcal {F}\subset \mathcal {O}(D)$, we say that the domain $D\subset \mathbb {C}^n$ is an $\mathcal {F}$ *-domain of holomorphy* if there are no domains $D_0,D_1\subset \mathbb {C}^n$ with $\emptyset \ne D_0\subset D_1\cap D$, $D_1\subsetneq D$ such that for any $f\in \mathcal {F}$ there exists an $\tilde{f}\in \mathcal {O}(D_1)$ with $\tilde{f} \equiv f$ on $D_0$.

Imitating the description of balanced $H^{\infty }$-domains of holomorphy (see e.g., [22]), we present an analogous description for balanced $L_h^2$-domains of holomorphy (in arbitrary dimension). The characterization given in Theorem 2 is expressed with the tools of extremal functions defined with the aid of homogeneous polynomials. As a consequence, the description delivers some kind of an approximation of homogeneous logarithmically plurisubharmonic functions with the help of logarithms of roots of absolute values of homogeneous polynomials. That kind of approximation extends to some degree classical results in more general or similar situations (see the final discussion in Sect. 2.2, [20, 22]).

The proof of Theorem 7 follows from results of Jucha (see [12]) where the problem of Wiegerinck was studied for Hartogs domains. Employing the potential theoretic methods applied by Jucha, we present additionally an effective and simple algebraic criterion for a homogeneous polynomial to be square integrable on pseudoconvex balanced domains in dimension two (see Theorem 11). This theorem has many consequences as to the positive definiteness of the Bergman kernel, and the positive definiteness of the Bergman metric is concerned (see Corollaries 12, 13, 15). In particular, with its help we may conclude the existence of domains of Siciak’s type having completely different properties (see Theorem 17). The fact that domains of Siciak’s type admits so different possibilities appear as a surprise to us.

A closely related problem to the study of the Bergman space is the theory of *the Bergman kernel* (we restrict our definition to that on the diagonal):

1 $$\begin{aligned} K_D(z):=\sup \{|f(z)|^2:f\in L_h^2(D),\;||f||\le 1\},\; z\in D, \end{aligned}$$

*the Bergman pseudometric* (defined for *D* such that $K_D(z)>0$, $z\in D$):

2 $$\begin{aligned} \beta _D^2(z;X):=\sum _{j,k=1}^n\frac{\partial ^2\log K_D}{\partial z_j\bar{\partial }z_k}(z)X_j\bar{X}_k,\;z\in D,\; X\in \mathbb {C}^n, \end{aligned}$$

and *the Bergman distance* $b_D$.

Although the problems of positive definiteness, being an $L_h^2$-domain of holomorphy or Bergman complete in the class of bounded balanced pseudoconvex domains, are either trivial or fully understood, the situation in the unbounded case is still unclear. We present a few results on these subjects. We think the methods and ideas presented in our paper may help the Reader to develop new methods to cope with the problems. Let us mention here that recently a lot of effort was invested in investigation of these problems in many classes of unbounded domains (see e.g., [1, 3–5, 17] or [18]). As we saw the problem of Wiegerinck drew a lot of effort recently and except for partial results already mentioned above, the problem was repeated in a recent survey on problems in the theory of several complex variables ([6]).

As already mentioned a special role in our considerations and motivation for understanding the phenomena in the class of unbounded balanced domains was played by a special class of (in some sense wild) domains. The example of that type was given by Siciak (see [21]), and it serves as a good candidate for counterexamples in many situations. Formally, the class $\mathcal {D}_S$ is the class of all pseudoconvex balanced domains satisfying the following properties: $D=D_h$ where $h\not \equiv 0$ and $h^{-1}(0)$ is dense in $\mathbb {C}^n$. Such domains exist in arbitrary dimension (see [21]).

## Around Balanced $L_h^2$-Domains of Holomorphy

### Preliminaries

Unless otherwise stated we shall work with

3 $$\begin{aligned} D:=D_h:=\{z\in \mathbb {C}^n:h(z)<1\} \end{aligned}$$

being a balanced domain, which means that $h:\mathbb {C}^n\rightarrow [0,\infty )$ is upper semicontinuous and homogeneous (i.e., $h(\lambda z)=|\lambda |h(z)$, $\lambda \in \mathbb {C}$, $z\in \mathbb {C}^n$). The fact that $D_h$ is pseudoconvex is equivalent to the plurisubharmonicity of $\log h$. To avoid some trivialities we often assume that $D\ne \mathbb {C}^n$ (equivalently, $h\not \equiv 0$).

As already mentioned the main interest of our paper is devoted to unbounded balanced domains. A natural class of such domains, which may be a good starting point for exploring properties of unbounded balanced domains, may be the class of * elementary balanced domains* by which we mean the family of domains defined by functions *h* given by the formula:

4 $$\begin{aligned} h(z):=|A^1 z|^{t_1}\cdot \ldots \cdot |A^N z|^{t_N},\; z\in \mathbb {C}^n, \end{aligned}$$

where *N* is a positive integer, $A^1,\ldots ,A^N:\mathbb {C}^n\rightarrow \mathbb {C}$ are non-zero linear mappings and the positive numbers $t_1,\ldots ,t_N>0$ satisfy $t_1+\cdots +t_N=1$.

In order to understand the situation in the class of unbounded balanced domains, it may be useful to understand how the situation looks like for elementary balanced domains. To work with a general unbounded balanced domain often requires more sophisticated methods to work with.

Denote by $\mathcal {H}^d$ the set of polynomials $P\in \mathbb {C}[z_1,\ldots ,z_n]$ that are homogeneous of degree *d* or identically equal to 0.

Define $$\mathcal {H}^d(D):=\mathcal {H}^d\cap L^2(D)$$.

One may find an orthonormal basis $\{P_{d,j}$: $d=0,1,\ldots , j\in \mathcal {J}_d\}$ of the space $L_h^2(D)$ where $P_{d,j}\in \mathcal {H}^d(D)$ (the sets of indices $\mathcal {J}_d$ are finite but may be also empty).

Then any $f\in \mathcal {O}(D)$ has the following expansion:

5 $$\begin{aligned} f(z)=\sum _{d=0}^{\infty }P_d(z), \end{aligned}$$

where $P_d\in \mathcal {H}^d$ are uniquely determined and the convergence is locally uniform. If, additionally, $f\in L^2(D)$, then $P_d=\sum _{j\in \mathcal {J}_d}\epsilon _{d,j}P_{d,j}$ and

6 $$\begin{aligned} ||f||^2=\sum _{d=0}^{\infty }||P_d||^2=\sum _{d=0}^{\infty }\sum _{j\in \mathcal {J}_d}|\epsilon _{d,j}|^2; \end{aligned}$$

here $\Vert \cdot \Vert$ denotes the $L^2_h$-norm.

With any $f\in \mathcal {O}(D)$ we may define a homogeneous function

7 $$\begin{aligned} h_f(z):=\limsup _{d\rightarrow \infty }\root d \of {|P_d(z)|},\;z\in \mathbb {C}^n. \end{aligned}$$

Observe that

8 $$\begin{aligned} f(\lambda z)=\sum _{d=0}^{\infty }P_d(z)\lambda ^d\in \mathbb {C}, \;z\in D, \lambda \in \mathbb {D}. \end{aligned}$$

Consequently, $h_f(z)\le 1$, $z\in D$, and thus $h_f\le h$.

One also gets the following equality

9 $$\begin{aligned} K_D(z)=\sum _{d=0}^{\infty }\sum _{j\in \mathcal {J}_d}|P_{d,j}(z)|^2,\; z\in D. \end{aligned}$$

Note that

10 $$\begin{aligned} \infty >K_D(\lambda z)=\sum _{d=0}^{\infty }\left( \sum _{j\in \mathcal {J}_d}|P_{d,j}(z)|^2\right) |\lambda |^{2d},\;\lambda \in \mathbb {D}, z\in \mathbb {C}^n, h(z)\le 1. \end{aligned}$$

Note that the expression $\sum _{j\in \mathcal {J}_d}|P_{d,j}(z)|^2$ gives the value of the reproducing kernel of the space $\mathcal {H}^d(D)$—it resembles the idea of approximating equilibrium measures as presented in [2] (see also [13]).

Consequently, we may define the following homogeneous function (note that the above representation shows that the function defined below does not depend on the choice of the orthonormal basis $\{P_{d,j}\}$)

11 $$\begin{aligned} h_{BK}(z):=\limsup _{d\rightarrow \infty }\root 2d \of {\sum _{j\in \mathcal {J}_d}|P_{d,j}(z)|^2},\; z\in \mathbb {C}^n. \end{aligned}$$

Note that $h_{BK}(z)\le 1$, $z\in D$. Therefore, $h_{BK}\le h$ on $\mathbb {C}^n$.

Let $f\in L_h^2(D)$ be represented as

12 $$\begin{aligned} f(z)=\sum _{d=0}^{\infty }P_d(z)=\sum _{d=0}^{\infty }\sum _{j\in \mathcal {J}_d}\epsilon _{d,j}P_{d,j}. \end{aligned}$$

Then the Schwarz inequality gives

13 $$\begin{aligned} |P_d(z)|\le \sqrt{\sum _{j\in \mathcal {J}_d}|\epsilon _{d,j}|^2}\sqrt{\sum _{j\in \mathcal {J}_d}|P_{d,j}(z)|^2}\le ||f||\sqrt{\sum _{j\in \mathcal {J}_d}|P_{d,j}(z)|^2}, \end{aligned}$$

from which we get the following inequality:

14 $$\begin{aligned} h_f\le h_{BK}. \end{aligned}$$

Consequently, we have

15 $$\begin{aligned} h_f^*\le h_{BK}^*\le h,\; f\in L_h^2(D). \end{aligned}$$

Recall that $u^*$, *u* a real-valued function, means the upper semicontinuous regularization of *u*.

### $L_h^2$-Domains of Holomorphy of Balanced Domains

Recall that the $L_h^2$-envelope of holomorphy of a balanced domain is univalent; even more, it is a balanced domain (see e.g., [10]). Therefore, for a balanced domain $D_h$ there is another (pseudoconvex) balanced domain $D_{\tilde{h}}$ such that $D_h\subset D_{\tilde{h}}$ (equivalently, $\tilde{h}\le h$), any $L_h^2$ function on *D* extends to an element from $\mathcal {O}(D_{\tilde{h}})$, and the domain $D_{\tilde{h}}$ is the largest one with this property.

We give now the precise description of the $L_h^2$-envelope of holomorphy (in other words the description of the function $\tilde{h}$) of a balanced domain.

#### Theorem 1

Let $D=D_h$ be a balanced domain in $\mathbb {C}^n$. Then its $L_h^2$-envelope of holomorphy is the domain $D_{h_{BK}^*}$.

#### Proof

Let $D_{\tilde{h}}$ be the $L_h^2$-envelope of holomorphy of *D*. First we show that $\tilde{h}\le h_{BK}^*$.

Take any $f\in L_h^2(D)$ and let $f=\sum _{d=0}^{\infty }\sum _{j\in \mathcal {J}_d}\epsilon _{d,j} P_{d,j}=\sum _{d=0}^{\infty }P_d$ with $\sum _{d=0}^{\infty }\sum _{j\in \mathcal {J}_d}|\epsilon _{d,j}|^2<\infty$. Then $|P_d|^2\le \sum _{j\in \mathcal {J}_d}|\epsilon _{d,j}|^2\sum _{j\in \mathcal {J}_d}|P_{d,j}|^2$. We claim that the series defining *f* converges locally uniformly on $\{h_{BK}^*<1\}$. In fact, take a compact $K\subset \{h_{BK}^*<1\}$. Then there is a $\theta \in (0,1)$ such that $h_{BK}^*(z)<1-2\theta$, $z\in K$. Consequently, the Hartogs lemma implies that $\root d \of {\sum _{j\in \mathcal {J}_{d,j}}|P_{d,j}(z)|^2}<1-\theta$, $z\in K$ and *d* big enough. Therefore, the series $\sum _{d=0}^{\infty }P_d$ defines a holomorphic function on $\{h_{BK}^*<1\}$ extending *f*. Thus $\tilde{h}\le h_{BK}^*$.

To finish the proof suppose that there is a $z_0\in \mathbb {C}^n$ such that $\tilde{h}(z_0)<h_{BK}^*(z_0)$. Then there is a $z_1$ such that

16 $$\begin{aligned} \tilde{h}(z_1)<\limsup _{d\rightarrow \infty }\root 2d \of {\sum _{j\in \mathcal {J}_d}|P_{d,j}(z_1)|^2}=h_{BK}(z_1)=1\le h(z_1). \end{aligned}$$

Consequently, for any $\epsilon >0$ the function

17 $$\begin{aligned} f:D\ni z\rightarrow \sum _{d=0}^{\infty }\sum _{j\in \mathcal {J}_d}\overline{P_{d,j}\left( \frac{z_1}{1+\epsilon }\right) }P_{d,j}(z) \end{aligned}$$

is from $L_h^2(D)$. To see this note that the series $\sum _{d=0}^{\infty }\sum _{j\in \mathcal {J}_d}\left| P_{d,j}\left( \frac{z_1}{1+\epsilon }\right) \right| ^2$ is convergent—it is sufficient to see that $\limsup _{d\rightarrow \infty }\root d \of {\sum _{j\in \mathcal {J}_d}\left| P_{d,j}\left( \frac{z_1}{1+\epsilon }\right) \right| ^2}=\frac{1}{(1+\epsilon )^2}<1$. The extendability of *f* to the domain $D_{\tilde{h}}$ implies that the function

18 $$\begin{aligned} \lambda \rightarrow f(\lambda z_1) \end{aligned}$$

extends to a holomorphic function on $\left\{ |\lambda |<\frac{1}{\tilde{h}(z_1)}\right\}$. But

19 $$\begin{aligned} f(\lambda z_1)=\sum _{d=0}^{\infty }\sum _{j\in \mathcal {J}_d}|P_{d,j}(z_1)|^2\frac{\lambda ^d}{(1+\epsilon )^d}, \end{aligned}$$

which would imply that $\frac{1}{1+\epsilon }=\limsup _{d\rightarrow \infty }\frac{\root d \of {\sum _{j\in \mathcal {J}_d}|P_{d,j}(z_1)|^2}}{1+\epsilon }\le \tilde{h}(z_1)$, which is however impossible for $\epsilon >0$ small. $\square$

Below we present a description of balanced $L_h^2$-domains of holomorphy.

#### Theorem 2

Let $D\subset \mathbb {C}^n$ be a balanced domain. Then the following are equivalent

(i) *D* is an $L_h^2$-domain of holomorphy,
(ii) $h(z)=h_f^*(z)$, $z\in \mathbb {C}^n$ for some function $f\in L_h^2(D)$,
(iii) $$h(z)=h_{BK}^*(z)$$, $$z\in \mathbb {C}^n$$.

#### Proof

The assumption that *D* is an $L_h^2$-domain of holomorphy implies the existence of an $f\in L_h^2(D)$ which does not extend through any boundary point (see e.g., [10]). Let us expand *f* in homogeneous polynomials $f=\sum _{d=0}^{\infty }P_d$. Suppose that there is a $z_0$ such that $h_f^*(z_0)=\left( \limsup _{d\rightarrow \infty }\root d \of {|P_d|}\right) ^*(z_0)<h(z_0)$. Without loss of generality we may assume that $h_f^*(z_0)<1=h(z^0)$. Then the definition of the regularization and the Hartogs Lemma imply that there is a neighborhood *U* of $z_0$ such that $|P_d(z)|<(1-\theta )^d$ for big *d* and $z\in U$ with a suitable $\theta \in (0,1)$. Then the function $\sum _{d=0}^{\infty }P_d$ converges locally uniformly in *U* and, consequently, *f* extends holomorphically through the boundary point $z_0$—a contradiction.

The equality $h\equiv h_f^*$ trivially implies the equality $h\equiv h_{BK}^*$.

The remaining implication is, by the assumption of the equality $h\equiv h_{BK}^*$, a direct consequence of the previous theorem. $\square$

Recall that for an arbitrary bounded pseudoconvex domain *D* the fact that it is an $L_h^2$-domain of holomorphy is equivalently described by the boundary behavior of its Bergman kernel (see [16]). We will extend one of these implications to the case of unbounded balanced domains.

#### Corollary 3

Let $D=D_h$ be balanced. Assume that

20 $$\begin{aligned} \limsup _{z\rightarrow z_0}K_D(z)=\infty ,\; \text { for any }z_0\in \partial D. \end{aligned}$$

Then *D* is the $L_h^2$-domain of holomorphy.

#### Proof

Suppose the opposite. Then there is a $z_0\in \partial D$ such that $h_{BK}^*(z_0)<1$. Then, as usual, there is a bounded neighborhood *U* of $z_0$ such that $\sum _{j\in \mathcal {J}_d}|P_{d,j}(z)|^2\le (1-\theta )^d$ for some $\theta \in (0,1)$, *d* large enough and $z\in U$. Consequently, $\sum _{d=0}^{\infty }\sum _{j\in \mathcal {J}_d}|P_{d,j}(z)|^2<M$, $z\in U$, for some $M<\infty$. This expression, however, gives the value of $K_D(z)$ for $z\in U\cap D$, which contradicts the assumption. $\square$

#### Remark 4

Observe that for an arbitrary domain with univalent envelope of holomorphy the condition in the above corollary implies that *D* is automatically pseudoconvex (see [14]).

It remains an open question whether any balanced $L^2_h$-domain of holomorphy satisfies this condition as it is the case for bounded domains.

It is intriguing that in many cases the upper regularization in Theorem 2 is not necessary (at least in the case of the function $h_{BK}$). To be more concrete, recall that pseudoconvex bounded balanced domains are always $L_h^2$-domains of holomorphy (see [9]). Or even more, making use of the Theorem of Takegoshi-Ohsawa ([15]) we get that for the bounded pseudoconvex balanced *D* we have (see [11])

21 $$\begin{aligned} \lim _{|\lambda |\rightarrow 1}K_D(\lambda z)=\infty ,\; h(z)=1. \end{aligned}$$

This implies that for a fixed *z* with $h(z)=1$ the series in (10) as the power series of the variable $|\lambda |$ has the radius of convergence equal to one (and thus $h_{BK}(z)=1$). So we may rewrite the above result as follows:

#### Proposition 5

Let $D=D_h$ be a bounded pseudoconvex balanced domain, i. e. *h* is logarithmically plurisubharmonic and $h^{-1}(0)=\{0\}$. Then we have the equality

22 $$\begin{aligned} h_{BK}(z)=h(z),\; z\in \mathbb {C}^n. \end{aligned}$$

#### Remark 6

It is unclear how to get Proposition 5 for arbitrary balanced $L_h^2$-domains of holomorphy (not necessarily bounded).

Let us once more emphasize that the above results except for their description of notions related with $L_h^2$-domain of holomorphy of balanced domains may also serve as results on approximation of homogeneous, logarithmically plurisubharmonic functions with the help of limits of functions involving homogeneous polynomials (see for instance Proposition 2.1, Theorems 3.1 and 3.2 in [22]) which constitute an example of classical type of results in the theory of plurisubharmonic functions.

## Bergman Spaces in Two-Dimensional Balanced Domains

Consider the following mapping

23 $$\begin{aligned} \Phi : \mathbb {C}^{n-1}\times (\mathbb {C}\setminus \{0\})\ni (z^{\prime },z_{n})\rightarrow \left( \frac{z^{\prime }}{z_n},z_n\right) . \end{aligned}$$

The above mapping when restricted to $D_h$ allows to reduce the problem of describing the dimension of the space $L_h^2(D_h)$ to that of $L_h^2(G_{\varphi })$ where

24 $$\begin{aligned} \varphi (w^{\prime }):=\log h(w^{\prime },1),\; G_{\varphi }:=\{w\in \mathbb {C}^n:|w_n|<\exp (-\varphi (w^{\prime }))\}. \end{aligned}$$

In other words in order to find $\dim L_h^2(D_h)$ it suffices to solve the same problem for a Hartogs domain with basis $\mathbb {C}^{n-1}$ and plurisubharmonic function $\varphi :\mathbb {C}^{n-1}\rightarrow [-\infty ,\infty )$ with the additional property $\limsup _{||w^{\prime }||\rightarrow \infty }(\varphi (w^{\prime })-\log ||w^{\prime }||)<\infty$ (obviously, here $\Vert \cdot \Vert$ is nothing than the Euclidean norm); we write that $\varphi \in \mathcal {L}$.

### Description of Balanced Domains in $\mathbb {C}^2$ with Trivial Bergman Spaces

Below we study the question of Wiegerinck for the class of balanced pseudoconvex domains in $\mathbb {C}^2$ applying results from [12].

Recall that

25 $$\begin{aligned} \limsup _{|\lambda |\rightarrow \infty }(\varphi (\lambda )-\log |\lambda |)=\limsup _{|\lambda |\rightarrow \infty }\log h\left( 1,\frac{1}{\lambda }\right) \le \log h(1,0)<\infty . \end{aligned}$$

Using the beginning of Section 2 from [12] this estimate immediately leads to $\triangle \varphi \le 2\pi$.

#### Theorem 7

Let $D=D_h$ be a balanced pseudoconvex domain in $\mathbb {C}^2$. Then $L_h^2(D)=\{0\}$ or it is infinitely dimensional. Moreover, $L_h^2(D)=\{0\}$ iff $h(z)=|Az|^t|Bz|^{1-t}$ or $h(z)=|Az|$ or $h(z)=0$, where *A*, *B* are non-trivial linear mappings $\mathbb {C}^2\rightarrow \mathbb {C}$ and $t\in (0,1)$.

#### Proof

Without loss of generality assume that $D\ne \mathbb {C}^2$. It follows from Theorem 4.1 in [12] that $L_h^2(D)=\{0\}$ iff $\varphi (\lambda )=\sum _j\alpha _j\log |\lambda -a_j|+g(\lambda )$, where $\alpha _j>0$, *g* is harmonic on $\mathbb {C}$, and some additional condition on the $\alpha _j$’s is satisfied. And in the case $L_h^2(D)$ is not trivial it is infinitely dimensional.

Recall that $\triangle \varphi \le 2\pi$. In particular, $\sum _j\alpha _j\le 1$. It also follows from the description of the triviality of $L_h^2(G_{\varphi })$ in Jucha’s paper (conditions on $\alpha _j$) that at most two $\alpha _j$’s are positive.

Then, by (25), we have

26 $$\begin{aligned} \limsup _{|\lambda |\rightarrow \infty }\left( \sum _j\alpha _j\log \left| 1-\frac{a_j}{\lambda }\right| +\left( \sum _j\alpha _j-1\right) \log |\lambda |+g(\lambda )\right) <\infty \end{aligned}$$

from which we conclude that

27 $$\begin{aligned} \limsup _{|\lambda |\rightarrow \infty }\left( \left( \sum _j\alpha _j-1\right) \log |\lambda |+g(\lambda )\right) <\infty . \end{aligned}$$

The above function is from $\mathcal {L}$, *g* is harmonic on $\mathbb {C}$, $\sum _j\alpha _j\le 1$ (and the sum is taken over a finite set). Thus, one easily gets from the standard properties of harmonic functions that $g\equiv const$ and $\sum _j\alpha _j=1$, which easily finishes the proof. $\square$

#### Remark 8

The above description of balanced domains with trivial Bergman space may be formulated as follows: $L_h^2(D)=\{0\}$ iff *D* is linearly equivalent to $\mathbb {C}^2$, $\mathbb {C}\times \mathbb {D}$ or $\{z\in \mathbb {C}^2:|z_1|^t|z_2|^{1-t}<1\}$ for some $t\in (0,1)$.

#### Remark 9

The above result leaves many questions open. For instance, does the dichotomy on the dimension of $L_h^2(D)$ remain true for pseudoconvex balanced domains in higher dimension?

What is the description of pseudoconvex balanced domains with a trivial Bergman space in higher dimension? Do they have to be elementary balanced domains as it is the case in dimension two?

Is every pseudoconvex balanced domain of holomorphy with non-trivial Bergman space an $L_h^2$-domain of holomorphy (as it is the case for the bounded domains)?

It follows from the above result that domains from the class $\mathcal {D}_S$ (in dimension two) always admit an infinitely dimensional Bergman space. Are these domains also $L_h^2$-domains of holomorphy?

#### Remark 10

Recall that the main result in [7] assures the infinite dimensionality of $L_h^2(D)$ in the case that the core $\mathfrak c^{\prime }(D)$ is different from *D*. Note that for the domains $D\in \mathcal {D}_S$ one has $\mathfrak c'(D)=D$. Therefore, the infinite dimensionality of $L^2_h(D)$ for these domains cannot be concluded from [7].

### Square Integrable Homogeneous Polynomials

Let $h:\mathbb {C}^2\rightarrow [0,\infty )$ be a logarithmically plurisubharmonic, homogeneous function with $h\not \equiv 0$.

For $[v]\in \mathbb {P}^1$ with $v_2\ne 0$ (the case when $v_1\ne 0$ is defined appropriately) define

28 $$\begin{aligned} \nu (\log h,[v]):=\nu (\log h(\cdot ,v_2),v_1), \end{aligned}$$

where $\nu (u,\cdot )$ denotes the Lelong number of a subharmonic function *u*. Note that the notion is well defined (it does not depend on the choice of a representative of [*v*] and the number of the chosen variable).

Recall that for a subharmonic function $u:D\rightarrow [-\infty ,\infty )$, $z_0\in D$ ($D\subset \mathbb {C}$ a domain) * the Lelong number is given as* (see e.g., [19]):

29 $$\begin{aligned} \nu (u,z_0):=\lim _{r\rightarrow 0}\frac{\frac{1}{2\pi }\int _0^{2\pi }u(z_0+re^{it})dt}{\log r}=\lim _{r\rightarrow 0}\frac{\max _{|\lambda |=r}u(z_0+\lambda )}{\log r}=\triangle u(\{z_0\}). \end{aligned}$$

We shall need the following properties of $\nu$ that will follow directly from that of the standard Lelong number:

- $$\sum _{[v]\in \mathbb {P}^1}\nu (\log h,[v])\le 1$$, $$[v]\in \mathbb {P}^1$$;
- $$\nu (t\log h_1+(1-t)\log h_2,\cdot )=t\nu (\log h_1,\cdot )+(1-t)\nu (\log h_2,\cdot )$$, $$t\in [0,1]$$.

For an element $[v]\in \mathbb {P}^1$ (with $v_2\ne 0$) and an $\epsilon >0$ we introduce the conic neighborhood of [*v*] in $\mathbb {C}^2$ as follows (in the case $v_2=0$ we may define it analogously with $v_1$ chosen):

30 $$\begin{aligned} U([v],\epsilon ):=\left\{ \frac{\mu }{v_2}(v_1,\lambda ):|\lambda -v_1|<\epsilon |v_2|,\lambda , \mu \in \mathbb {C}\right\} . \end{aligned}$$

For $0\not \equiv Q\in \mathcal {H}^d$ we calculate (assuming that $v_2\ne 0$):

31 $$\begin{aligned}&\int _{\{h<1\}\cap U([v],\epsilon )}|Q(z)|^2d\mathcal {L}^4(z)\nonumber \\&\quad =\int _{\{w_1:|w_1-v_1|<\epsilon |v_2|\}}\left( \int _{\{w_2:|w_2|<\exp (-\varphi (w_1))\}}|Q(w_1,1)|^2|w_2|^{2d+2}d\mathcal {L}^2(w_2)\right) d\mathcal {L}^2(w_1)\nonumber \\&\quad =\frac{\pi }{d+2}\int _{\{w_1:|w_1-v_1|<\epsilon |v_2|\}}|Q(w_1,1)|^2\exp \left( -2(d+2)\varphi (w_1)\right) d\mathcal {L}^2(w_1). \end{aligned}$$

It follows from properties of the Lelong numbers (see Corollary 2.4(a) from [12]) that the last expression is finite (for sufficiently small $\epsilon >0$) iff

32 $$\begin{aligned} m(Q,[v])>\nu (\log h,[v])(deg Q+2)-1. \end{aligned}$$

Here *m*(*Q*, [*v*]) denotes the multiplicity of the zero of the homogeneous polynomial by which we mean the multiplicity of the zero of the polynomial $Q(v_1,\lambda )$ at $\lambda =v_2$ (if $v_1\ne 0$) or the multiplicity of the polynomial $Q(\lambda ,v_2)$ at $\lambda =v_1$ (if $v_2\ne 0$).

Making use of the standard compactness argument, we get the following.

#### Theorem 11

Fix $n=2$. Let $Q\in \mathcal {H}^d$ and let $h:\mathbb {C}^2\rightarrow [0,\infty )$ be a logarithmically plurisubharmonic homogeneous function. Then $Q\in L_h^2(D_h)$ if

33 $$\begin{aligned} m(Q,[v])>\nu (\log h,[v])(d+2)-1\; \text { for any } [v]\in \mathbb {P}^1. \end{aligned}$$

Now, the above result has many consequences.

#### Corollary 12

For the balanced pseudoconvex domain $D_h\subset \mathbb {C}^2$ the following are equivalent:

(i) $$K_D(0)>0$$;
(ii) $$K_D(z)>0$$, $$z\in D$$;
(iii) $$\mathcal {L}^4(D_h)<\infty $$;
(iv) $$\nu (\log h,[v])<1/2$$, $$[v]\in \mathbb {P}^1$$.

#### Proof

Recall that for the balanced pseudoconvex domain we get $K_D(0)=\frac{1}{\mathcal {L}^4(D)}$, which easily gives the equivalence of the first three conditions equivalent. Theorem 11 applied to $Q\equiv 1$ gives the equivalence of its integrability to the last condition. $\square$

Similarly, we get the following.

#### Corollary 13

For the balanced pseudoconvex domain $D_h\subset \mathbb {C}^2$ the following are equivalent:

(i) $D_h$ admits the Bergman metric;
(ii) the functions $z_1,z_2\in L_h^2(D_h)$;
(iii) $$\nu (\log h,[v])<1/3$$, $$[v]\in \mathbb {P}^1$$.

#### Proof

The equivalence of the second and third condition follows from Theorem 11 (applied to the functions $z_1$ and $z_2$). It $D_h$ admits the Bergman metric, then the positive definiteness of the Bergman metric at 0 easily implies that $z_1,z_2$ are square integrable. On the other hand, assuming the third property we get from the previous corollary the positive definiteness of the Bergman kernel. The fact that in this the functions $z_1,z_2$ are square integrable follows from Theorem 11 which finishes the proof. $\square$

#### Example 14

To illustrate the results from above we give the following three simple examples:

1. $$h(z,w):=|zw|^{1/2}$$. Then $$K_{D_h}(0)=0$$.
2. $h(z,w):=|z(z-w)w|^{1/3}$. Then $K_{D_h}(0)>0$, but $D_h$ admits no Bergman metric.
3. $h(z,w):=|z(z-w)(z+w)w|^{1/4}$. Then $D_h$ admits a Bergman metric.

#### Corollary 15

Let $D_h\subset \mathbb {C}^2$ be a balanced pseudoconvex domain. Then $L_h^2(D_h)$ contains all the polynomials iff $\nu (\log h,[v])=0$ for all $[v]\in \mathbb {P}^1$.

#### Remark 16

Recall that there are balanced (in fact even Reinhardt) domains that are not bounded but that satisfy the assumption of the previous corollary. In fact, we may take for instance

34 $$\begin{aligned} D:=\left\{ z\in \mathbb {C}^2:|z_2|<1 \text { and } |z_2|<\exp (-(\log |z_1|)^2) \text { for } |z_1|\ge 1\right\} . \end{aligned}$$

The fact that all the monomials belong to $L_h^2(D)$ may be verified either by the direct estimates of the appropriate integrals or by applying the description of square integrable monomials in pseudoconvex Reinhardt domains that may be found in [25] or [26].

It turns out that there are domains from $\mathcal {D}_S$ having quite different properties as its connection with the Bergman theory is concerned. First recall that we have already seen that all such domains have their $L_h^2$ space infinitely dimensional. Additionally, we have the following

#### Theorem 17

(i) There is a $D\in \mathcal {D}_S$ such that its four-dimensional volume $\mathcal {L}^4(D)=\infty$.

(ii) There is a $D\in \mathcal {D}_S$ such that $K_D(z)>0$, $z\in D$ (equivalently, $\mathcal {L}^4(D)<\infty$) but *D* does not admit the Bergman metric.
(iii) There is a $D\in \mathcal {D}_S$ such that *D* admits the Bergman metric.

#### Proof

Let $D=D_{h_0}$ be some domain from $\mathcal {D}_S$. Consider functions $h\ge 0$ such that $\log h$ is of the form

35 $$\begin{aligned} \log h=t \log h_0(z)+(1-t)\frac{1}{n}\sum _{j=1}^n\log |z_1-a_jz_2|, \end{aligned}$$

where $t\in (0,1)$ and $a_1,\ldots a_n\in \mathbb {C}$, such that $[(1,a_1)],\ldots ,[(1,a_n)]\in \mathbb {P}^1$, satisfy $\nu (\log h,[(1,a_j)])=0$ for $j=1,\ldots ,n$.

It is now a straightforward consequence of the above corollaries, that, no matter how $h_0$ was chosen, one can arrange, after a suitable choice of *t* and *n*, that $D_h$ has one of the properties listed in the theorem. $\square$

#### Remark 18

Examples that were studied in [17] show that it is a kind of the Lelong number which may be responsible for the fact that the balanced domain is Bergman complete. More precisely, the following description of Bergman complete balanced domains in $\mathbb {C}^2$ may be correct:

$D=D_h$ is Bergman complete iff $\nu (\log ,[v])=0$ for any $[v]\in \mathbb {P}^1$.

Recall that all bounded pseudoconvex balanced domains are Bergman complete (see [11]) as well as the domain defined in (34) (see [17]).

As we have seen the domains from the class $\mathcal {D}_S$ may have quite different properties. But does there exist a two-dimensional domain $D=D_h\in \mathcal {D}_S$ such that $\nu (\log ,[v])=0$ for any $[v]\in \mathbb {P}^1$?
